# Differences in Striatal Metabolism in [^18^F]FDG PET in Parkinson’s Disease and Atypical Parkinsonism

**DOI:** 10.3390/diagnostics13010006

**Published:** 2022-12-20

**Authors:** Alexander P. Seiffert, Adolfo Gómez-Grande, Laura Alonso-Gómez, Antonio Méndez-Guerrero, Alberto Villarejo-Galende, Enrique J. Gómez, Patricia Sánchez-González

**Affiliations:** 1 Biomedical Engineering and Telemedicine Centre, ETSI Telecomunicación, Center for Biomedical Technology, Universidad Politécnica de Madrid, 28040 Madrid, Spain; 2 Department of Nuclear Medicine, Hospital Universitario 12 de Octubre, 28041 Madrid, Spain; 3 Facultad de Medicina, Universidad Complutense de Madrid, 28040 Madrid, Spain; 4 Department of Neurology, Hospital Universitario 12 de Octubre, 28041 Madrid, Spain; 5 Group of Neurodegenerative Diseases, Hospital 12 de Octubre Research Institute (imas12), 28041 Madrid, Spain; 6 Biomedical Research Networking Center in Neurodegenerative Diseases (CIBERNED), 28029 Madrid, Spain; 7 Centro de Investigación Biomédica en Red de Bioingeniería, Biomateriales y Nanomedicina, Instituto de Salud Carlos III, 28029 Madrid, Spain

**Keywords:** neuroimaging, [^18^F]FDG PET, neurodegenerative diseases, Parkinson’s disease, atypical parkinsonism, striatum

## Abstract

Neurodegenerative parkinsonisms affect mainly cognitive and motor functions and are syndromes of overlapping symptoms and clinical manifestations such as tremor, rigidness, and bradykinesia. These include idiopathic Parkinson’s disease (PD) and the atypical parkinsonisms, namely progressive supranuclear palsy (PSP), corticobasal degeneration (CBD), multiple system atrophy (MSA) and dementia with Lewy body (DLB). Differences in the striatal metabolism among these syndromes are evaluated using [^18^F]FDG PET, caused by alterations to the dopaminergic activity and neuronal loss. A study cohort of three patients with PD, 29 with atypical parkinsonism (10 PSP, 6 CBD, 2 MSA, 7 DLB, and 4 non-classifiable), and a control group of 25 patients with normal striatal metabolism is available. Standardized uptake value ratios (SUVR) are extracted from the striatum, and the caudate and the putamen separately. SUVRs are compared among the study groups. In addition, hemispherical and caudate-putamen differences are evaluated in atypical parkinsonisms. Striatal hypermetabolism is detected in patients with PD, while atypical parkinsonisms show hypometabolism, compared to the control group. Hemispherical differences are observed in CBD, MSA and DLB, with the latter also showing statistically significant caudate–putamen asymmetry (*p* = 0.018). These results indicate disease-specific metabolic uptake patterns in the striatum that can support the differential diagnosis.

## 1. Introduction

Neurodegenerative diseases are a group of diseases characterized by the progressive degeneration or death of neurons and are a major cause of mortality and morbidity. Distinctions can be made at a molecular level and the associated syndromes present different clinical manifestations with characteristic symptoms and affect specific cognitive or motor functions [1]. However, overlaps of the main features may occur, which is the case of the parkinsonian syndromes or parkinsonisms. Idiopathic Parkinson’s disease (PD) is the second most common neurodegenerative disease after Alzheimer’s disease [2]. It is characterized by the degeneration of dopaminergic neurons in the substantia nigra and the nigrostriatal pathway resulting in an impairment of motor functions including tremor, rigidness and bradykinesia [3]. These symptoms can also be present in atypical parkinsonism, which include progressive supranuclear palsy (PSP), corticobasal degeneration (CBD), multiple system atrophy (MSA) and dementia with Lewy body (DLB) [4,5]. The accurate differential diagnosis of parkinsonisms is important as the prognosis and treatment response is different in each case [6].

Due to the progressive degeneration of dopaminergic neurons and decrease of dopamine transporter (DaT) density, both SPECT and PET pre and postsynaptic dopaminergic imaging are used for the differential diagnosis [7]. Functional imaging of the dopaminergic activity such as [^123^I]FP-CIT SPECT (DaTSCAN) or [^18^F]fluorodopa PET imaging has shown clinical use for discriminating neurodegenerative parkinsonisms from other types such as vascular or drug-induced parkinsonism, or essential tremor [7,8]. However, the presynaptic alterations present in PD cannot be differentiated of the postsynaptic alterations in atypical parkinsonisms using these images [7,9]. In contrast, typical from atypical parkinsonisms can be differentiated by postsynaptic imaging (e.g., [^123^I]IBZM SPECT, and lately [^18^F]fluorodeoxyglucose ([^18^F]FDG) PET imaging), where PD shows increased and atypical parkinsonism decreased uptake [7,9,10]. However, postsynaptic imaging does not allow for distinguishing among atypical parkinsonisms, and it is not included in the diagnostic criteria or used in clinical routine practice.

[^18^F]FDG PET imaging is one of the main tools used in the evaluation of neurodegenerative diseases, especially for the differential diagnosis of Alzheimer’s disease (AD) [11]. As such, it is a biomarker of neurodegeneration presenting disease-specific patterns of hypometabolism, e.g., parietotemporal hypometabolism in AD [12,13]. In the same way, PD and atypical parkinsonism also present disease-specific patterns of altered metabolism. Eidelberg et al. [14] identified a PD-related pattern, which was replicated by Meles et al. [15], of hypermetabolism in the thalamus, pallidus/putamen, pons, and cerebellum, and hypometabolism in cortical regions such as the frontal, occipital and parietal cortices. In the case of atypical parkinsonisms, and its differentiation from PD, [^18^F]FDG PET has shown to be useful and with distinct patterns [6,16,17,18,19,20,21,22]. Reduced striatal metabolism is the most consistent feature differentiating PD from atypical parkinsonisms due to the post-synaptic loss in these cases [19].

In this paper, the metabolic activity of the striatum in patients with PD and atypical parkinsonism is studied. The overall activity compared to a control group is evaluated and patterns of intrastriatal differences in atypical parkinsonisms are analyzed. Currently, metabolic imaging is used in clinical routine practice for the differential diagnosis based on cortical uptake values, but the striatum is not interpreted. The aim of the study was to evaluate the value of semi-quantitative analysis of the striatum in [^18^F]FDG PET imaging for the differential diagnosis of PD and atypical parkinsonism.

## 2. Materials and Methods

### 2.1. Patients

Patients that underwent a [^18^F]FDG PET/CT scan at the Department of Nuclear Medicine of the Hospital Universitario 12 de Octubre, Madrid, Spain, between 2015 and 2021 were eligible for the study cohort. Patients with potential diagnoses of PD and atypical parkinsonism (PSP, CBD, MSA, DLB or non-classifiable (NC)) in neurological and nuclear medicine reports were included. All reports were reviewed by experienced specialists in neurology and nuclear medicine to confirm the diagnoses or exclude the patients. PD patients with visually and Z-score-based normalized striatal metabolism in [^18^F]FDG PET images were excluded.

Additionally, a control group of patients with normal striatal metabolism was defined from patients that underwent amyloid PET/CT imaging between 2014 and 2021 at the Department of Nuclear Medicine of the Hospital Universitario 12 de Octubre, Madrid, Spain. The following exclusion criteria were defined: (1) cognitive stages different than Mild Cognitive Impairment (MCI) and without any movement disorder symptoms, (2) patients without [^18^F]FDG PET scans, (3) [^18^F]FDG PET scans with at least one cortical region presenting hypometabolism defined as a Z-score below −2, and (4) [^18^F]FDG PET scans with cortical regions presenting a positive or very high Z-score. Z-scores were obtained using the CortexID Suite (GE Healthcare, Chicago, IL, USA).

### 2.2. Image Acquisition

Prior to [^18^F]FDG injection, patients underwent a 6 h fasting period, and were advised to drink water and urinate before image acquisition. Images were acquired with a Biograph 6 True Point PET/CT scanner (Siemens Healthineers, Erlangen, Germany) or with a Discovery MI DR PET/CT scanner (GE Healthcare, Chicago, Illinois), with both scanners being equivalently calibrated. A mean dose of 207 ± 59 MBq of [^18^F]FDG was injected intravenously. Images were acquired between 20 and 81 min after radiotracer injection and scan duration was 30 min. Reconstructed images had a matrix size of 336 × 336 × 55 and voxel size of 1.01821 × 1.01821 × 3 mm^3^.

### 2.3. Image Analysis

**Figure 1:**
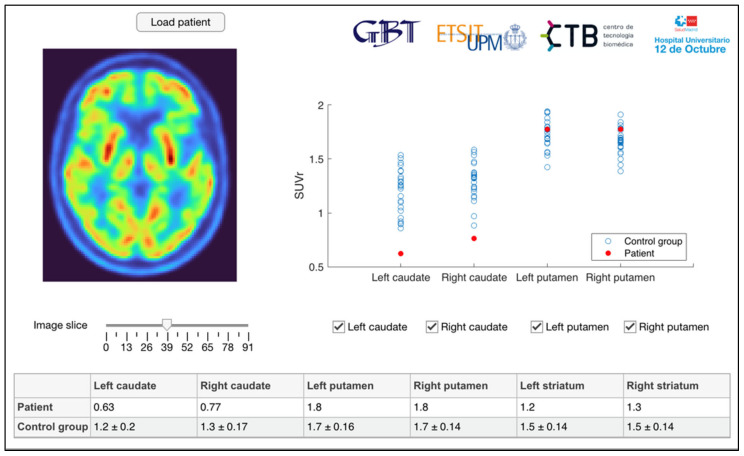
Processing and visualization application.

All [^18^F]FDG PET images are preprocessed using SPM12 (http://www.fil.ion.ucl.ac.uk/spm/ (accessed on 1 December 2022)) and segmentation and quantification is performed in MATLAB (The MathWorks Inc., Natick, MA, USA) [13]. Images are oriented manually in native space, PET images are coregistered to the corresponding CT images, and spatially normalized to a standard space defined by the Montreal Neurological Institute (MNI) using the CT image as anatomical reference following [23]. The resulting images have a matrix size of 91 × 109 × 91 and voxel size of 2 × 2 × 2 mm^3^. Atlases provided by PMOD (PMOD Technologies, Zurich, Switzerland) and based on the Automatic Anatomical Labelling atlas [24] are used to segment a total of seven regions of interest (ROI), corresponding to the left and right caudate and putamen. Caudate and putamen masks are also combined for striatal ROIs, creating one for each hemisphere and an additional combined striatal ROI. Standardized Uptake Value Ratios (SUVR) are calculated normalizing the mean image intensities of the ROIs by the mean intensity of a pons reference region. Image processing and visualization was performed using an application developed in-house [13] inspired by the DaTQUANT^TM^ software by GE Healthcare (see Figure 1) [25].

### 2.4. Statistical Analysis

Quantitative variables are represented as mean ± standard deviation (SD). The normality of the distributions is tested using the Shapiro–Wilk test. Differences in striatal [^18^F]FDG uptake among PD, atypical parkinsonisms and patients with visually normal striatal uptake, as well as among the specific atypical parkinsonisms, are evaluated by one-way ANOVA or Kruskal–Wallis tests and *post hoc* pairwise comparisons with Bonferroni adjusted α values. Additionally, hemispherical differences of metabolic activity in the caudate and putamen, as well as differences between these two regions per hemisphere, are analyzed by paired *t*-tests or Wilcoxon signed rank tests for each atypical parkinsonism-specific group. Lastly, multinomial logistic regression is used based on caudate and putamen SUVRs to model the diagnosis of PD, atypical parkinsonism, and control, as well as the diagnosis of the different types of atypical parkinsonism. Statistical analysis is performed using SPSS software version 26.00 (IBM Corp., Armonk, NY, USA), and *p*-values < 0.05 are considered statistically significant.

## 3. Results

### 3.1. Study Cohort

The study cohort consisted initially of a total of 44 patients diagnosed with PD or atypical parkinsonism. After reviewing the medical records and [^18^F]FDG PET images, a total of eight patients were removed. Three of these eight patients were transferred to the control group due to no diagnosis of a neurodegenerative disease, and five were excluded due to diagnoses of other diseases like AD, aphasia, or primary lateral sclerosis. In addition, five patients diagnosed with PD with normalized striatal metabolism due to medication at the time of the image acquisition were excluded. Finally, the study cohort consisted of 32 patients, 3 with PD and 29 with atypical parkinsonism. Specifically, 10 patients with PSP, 6 with CBD, 2 with MSA, 7 with DLB and 4 NC were included. Regarding the control group, a total of 25 patients were included after applying the previously defined exclusion criteria to an initial database of 175 patients. Patient demographics are summarized in Table 1.

### 3.2. Comparison of PD, Atypical Parkinsonism and Control

SUVRs of the striatum and its subregions (caudate and putamen) show statistically significant differences among PD, atypical parkinsonism, and control patients (Table 2). A general pattern in all ROIs can be observed where PD presents hypermetabolism (Striatum SUVR: 1.57 ± 0.01) compared to the control group (striatal SUVR: 1.48 ± 0.13) while the atypical parkinsonisms present the lowest striatal metabolism (striatal SUVR: 1.27 ± 0.14). *Post hoc* analyses (Table 3) revealed statistically significant differences between PD and atypical parkinsonisms, and atypical parkinsonisms and the control group, in all ROIs except the right caudate (*p* = 0.059).

Regarding the multinomial logistic regression analysis, the model was statistically significant and is able to predict the diagnosis based on caudate and putamen SUVRs (χ^2^(8) = 40.096, *p* < 0.001). PD is predicted with an accuracy of 33.3%, atypical parkinsonism with 86.2% and control with 88.0%. The overall accuracy is 84.2%.

### 3.3. Comparison of Atypical Parkinsonisms

Following the grouping of the different types of atypical parkinsonism into one group, differences in the striatal metabolism among these types are analyzed separately. Table 4 summarizes the striatal and subregional SUVRs, as well as the corresponding *p*-values. Statistically significant differences can be observed in the caudate (left: *p* = 0.034, right: *p* = 0.010), and the striatum (left: *p* = 0.027, right: *p* = 0.008 and, whole: *p* = 0.035, respectively). Overall, the highest SUVRs can be observed in patients with MSA (striatal SUVR: 1.50 ± 0.02), while the lowest are present in the NC group (striatal SUVR: 1.11 ± 0.13). *Post hoc* analyses (see Table S1 of the Supplementary Material) show statistically significant differences between MSA and NC groups in the caudate (left: *p* = 0.035, right: *p* = 0.011) and the striatum (left: *p* = 0.037, right: *p* = 0.008).

Hemispherical differences are studied for all three regions, and the results are summarized in Table 5. In the caudate, lower SUVRs in the left hemisphere can be observed for all types of atypical parkinsonism except the NC group, while being statistically significant in the CBD (*p* = 0.003), MSA (*p* <0.001) and DLB (*p* = 0.001) groups. In contrast, the putamen shows higher SUVRs in the left hemisphere than the right, with the differences being statistically significant in all Parkinsonism types. Following the pattern of the caudate, in the striatum, the SUVRs are again lower in the left hemisphere than the right. Statistically significant differences can again be observed in the CBD (*p* = 0.002), MSA (*p* < 0.001), and DLB (*p* = 0.004) groups.

Metabolic differences between the caudate and the putamen in the same hemisphere are evaluated, and the results are summarized in Table 6. Overall, SUVRs are lower in the caudate. Statistically significant differences can only be observed in the DLB group (left: *p* = 0.018, right: *p* = 0.018).

Lastly, a multinomial logistic regression model is generated based exclusively on the caudate and putamen SUVRs to predict the type of atypical parkinsonism. The model is statistically significant (χ^2^(8) = 30.929, *p* = 0.014) with an overall accuracy of 55.2%. It predicts PSP, CBD, MSA, DLB and NC with accuracies of 80.0%, 33.3%, 100.0%, 14.3% and 75.0%, respectively.

## 4. Discussion

In this study, [^18^F]FDG PET images are semi-quantitatively analyzed to assess the striatal metabolism in three patients with PD and 29 patients with atypical parkinsonism, and among types of atypical parkinsonism. Potential distinct patterns of hypermetabolism in PD patients, as well as hypometabolism in those with atypical parkinsonism are identified. In addition, different types of atypical parkinsonism show different patterns of intrastriatal metabolism with either hemispherical differences or between the caudate and the putamen. Multinomial logistic regression analyses show statistically significant models to predict the diagnosis based exclusively on the striatal metabolism.

The increased metabolism in the PD group can be observed in both the caudate and the striatum compared to the control group, without hemispherical differences. However, the differences were not statistically significant. In the same way, various studies [15,16,26] found bilateral hypermetabolism in the putamen while Teune et al. [18] described increased metabolism in the putamen contralateral to the affected body side. Compared to atypical parkinsonisms, striatal metabolism, except in the right caudate, was significantly increased in PD. In addition, the striatal metabolism in atypical parkinsonisms was also significantly lower than in the control group, demonstrating the potential of [^18^F]FDG PET for detecting these three groups. In PD, postsynaptic activity is increased, as evidenced by SPECT and PET imaging with dopamine D2-receptor-binding radiotracers, while it is reduced in atypical parkinsonism [7,9,10]. This same pattern is present in our results with metabolic imaging, and it has been suggested that [^18^F]FDG PET is a marker of postsynaptic activity [27].

Among atypical parkinsonisms, the striatal differences in metabolic activity were less pronounced. Overall differences in *post hoc* analyses were only present between MSA and NC, and, in nearly all cases, statistically significant hemispherical differences were found. In PSP, asymmetry was shown by Teune et al. [18] and Hellwig et al. [28] but not by Eckert et al. [16] and our study where the hypometabolism was bilateral in the caudate (*p* = 0.263). In CBD, the various previous studies found asymmetry in the basal ganglia [16,18,28]. Eckert et al. [16] and Teune et al. [18] described bilateral hypometabolism for the putamen in MSA, which in our study cohort presents the second highest metabolism out of the atypical parkinsonisms, albeit more similar to that of the caudate than in the other cases. In contrast, a statistically significant difference between caudate and putamen metabolism could only be observed in DLB (*p* = 0.018). This asymmetry was also described by Hellwig et al. [28]; however, the putamen showed hypermetabolism, while, in our study, it is significantly higher than in the caudate but below the control group. Given the similarity of the striatal metabolism in some types of atypical parkinsonisms, the concomitant evaluation with cortical uptake patterns is recommended, and [^18^F]FDG PET has also been shown to better discriminate atypical parkinsonisms than postsynaptic [^123^I]IBZM SPECT imaging [28].

Most of the obtained results are consistent with the scientific literature; however, the diagnoses, while reviewed for this study, were not confirmed, and incorrect classifications would alter the expected striatal metabolism. In addition, artifactual increases or decreases of the striatal SUVRs may be caused by considerable alterations in the reference region of the patients of the study cohort. Even though the pons has previously been used for intensity normalization in PD [29], it is also included in the PD-related metabolic pattern as hypermetabolic [14,15]. Moreover, no statistically significant differences among pontine uptakes have been observed in atypical parkinsonisms in our study cohort (*p* = 0.631). In contrast, Albrecht et al. [30] described in their meta-analysis that subcortical hypermetabolism was found in studies using global mean normalization but was absent in those using white matter, cerebellum, pons, or absolute measures, which may be caused by decreased grey matter metabolism as is identified for PD [14,15]. 

As described above regarding the study cohort, more than half of the patients with PD had to be excluded due to possible decreases of the striatal metabolic activity by levodopa treatment following a review of the clinical reports. In these patients, left and right striatal SUVRs were 1.32 ± 0.14 (Caudate: 0.97 ± 0.17, Putamen: 1.66 ± 0.18) and 1.37 ± 0.11 (Caudate: 1.11 ± 0.13, Putamen: 1.62 ± 0.15), respectively. In all regions, SUVRs show values that are more akin to those of the control group or even show hypometabolism as is the case of the atypical parkinsonisms (see Table 2). Decreased cerebral metabolism for patients treated with levodopa has also been shown in previous studies [31,32,33]. In the remaining three cases included in the study cohort, prior to image acquisition, medication had been initiated only a few days prior to the image acquisition, the patient had stopped, or it did not have the expected effect.

Regarding the limitations of this study, the limited number of patients composing the final study group needs to be mentioned. On one hand, only three patients could be included with a diagnosis of PD after excluding those with normalized striatal metabolism after levodopa treatment. In the case of the atypical parkinsonisms, the same occurred for the MSA group. Due to the retrospective nature of the study and the importance of DaT imaging in the diagnosis, only a few [^18^F]FDG PET scans were available for patients with PD or atypical parkinsonism. In addition, only patients with atypical clinical manifestations, i.e., doubtful cases, were transferred for [^18^F]FDG PET imaging, resulting in a small study cohort. The control group was also composed not of healthy cognitively normal subjects but patients with normal striatal uptake but who may present cortical metabolic and cognitive alterations. In addition, the patients of the control group were significantly younger than those of the PD and atypical parkinsonism groups (Age (years): 63.84 ± 8.23 vs. 73.28 ± 8.45, *p* < 0.001), which may be expected due to the diagnoses of MCI in the control group. We also specifically excluded those patients from the control group with any movement disorder symptoms when presenting normal striatal uptake, considering symptomatology to be a more important criterion than age when defining both groups. While age-related effects on metabolic activity and [^18^F]FDG distribution have been shown in subcortical regions such as the striatum, it is more pronounced in cortical regions and has been observed to be more stable until 60–70 years in some regions, corresponding approximately to the age bracket of our study [34,35]. Therefore, the results concerning these groups should be taken with caution. In future studies, the database should be expanded, especially to balance the different groups of atypical parkinsonism, to include approximately the same number of patients with Parkinson’s disease, and to define the control group more strictly regarding cognitive stage and age. Moreover, additional machine learning algorithms with feature selection and hyperparameter optimization should be evaluated to improve the classification performance. Lastly, it is proposed to further investigate the effect of treatment on striatal metabolism, as in this study, it is shown to reduce the hyperactivity usually present in the [^18^F]FDG PET images of PD patients.

## 5. Conclusions

PD and atypical parkinsonisms are neurodegenerative diseases characterized by alterations to the DaT activity in the striatum. By semi-quantitatively analyzing [^18^F]FDG PET imaging, PD patients showed hypermetabolism compared to a control group, while hypometabolism was characteristic in atypical parkinsonisms. In addition, some differences in striatal metabolism were found for different types of atypical metabolism. Striatal metabolism was able to accurately predict the differential diagnosis.

## Figures and Tables

**Table 1:** Patient demographics of final study cohort.

Diagnosis	N	Age (Years ± SD)	Sex (m/f)
PD	3	74.33 ± 11.72	1/2
Atypical parkinsonism	29	73.28 ± 8.45	17/12
PSP	10	71.80 ± 7.94	9/1
CBD	6	74.00 ± 7.40	5/1
MSA	2	69.50 ± 10.61	0/2
DLB	7	75.43 ± 9.20	2/5
NC	4	74.00 ± 12.25	1/3
Control group	25	63.84 ± 8.23	11/14

**Table 2:** Striatal SUVRs of the study groups.

Region		PD	Atypical Parkinsonism	Control Group	*p*-Value
Caudate	L	1.29 ± 0.03	0.89 ± 0.23	1.20 ± 0.20	<0.001
	R	1.32 ± 0.03	0.98 ± 0.28	1.29 ± 0.17	<0.001
Putamen	L	1.84 ± 0.05	1.60 ± 0.14	1.75 ± 0.16	0.001
	R	1.81 ± 0.05	1.56 ± 0.17	1.68 ± 0.14	0.003
Striatum	L	1.57 ± 0.02	1.25 ± 0.13	1.48 ± 0.14	<0.001
	R	1.57 ± 0.02	1.28 ± 0.16	1.49 ± 0.13	<0.001
	Whole	1.57 ± 0.01	1.27 ± 0.14	1.48 ± 0.13	<0.001

**Table 3:** Results of *post hoc* analyses comparing striatal SUVRs.

Region		PD—Atypical Parkinsonism	PD—Control Group	Atypical Parkinsonism—Control Group
Caudate	L	0.011	1.000	<0.001
	R	0.059	1.000	<0.001
Putamen	L	0.034	0.983	0.002
	R	0.025	0.475	0.017
Striatum	L	0.001	0.802	<0.001
	R	0.005	1.000	<0.001
	Whole	0.001	0.874	<0.001

**Table 4:** Striatal SUVRs of types of atypical parkinsonism.

Region		PSP	CBD	MSA	DLB	NC	*p*-Value
Caudate	L	0.92 ± 0.14	0.94 ± 0.23	1.26 ± 0.27	0.80 ± 0.28	0.68 ± 0.10	0.034
	R	1.05 ± 0.19	1.04 ± 0.24	1.44 ± 0.06	0.90 ± 0.26	0.64 ± 0.19	0.010
Putamen	L	1.56 ± 0.45	1.67 ± 0.17	1.66 ± 0.09	1.59 ± 0.14	1.59 ± 0.15	0.690
	R	1.52 ± 0.13	1.63 ± 0.18	1.61 ± 0.30	1.58 ± 0.17	1.49 ± 0.20	0.713
Striatum	L	1.25 ± 0.08	1.31 ± 0.16	1.46 ± 0.09	1.21 ± 0.12	1.14 ± 0.10	0.027
	R	1.29 ± 0.12	1.35 ± 0.16	1.53 ± 0.13	1.25 ± 0.10	1.08 ± 0.18	0.008
	Whole	1.27 ± 0.09	1.33 ± 0.16	1.50 ± 0.02	1.23 ± 0.11	1.11 ± 0.13	0.035

**Table 5:** Hemispherical differences in atypical parkinsonisms (*p*-values).

Atypical Parkinsonism	Caudate	Putamen	Striatum
PSP	0.263	<0.001	0.197
CBD	0.003	0.007	0.002
MSA	<0.001	<0.001	<0.001
DLB	0.001	0.001	0.004
NC	0.592	0.044	0.190

**Table 6:** Differences between caudate and putamen in atypical parkinsonisms (*p*-values).

Atypical Parkinsonism	Caudate–Putamen	
	L	R
PSP	0.278	0.735
CBD	0.531	0.634
MSA	0.180	0.655
DLB	0.018	0.018
NC	0.068	0.261

## Data Availability

The data presented in this study are available on request from the corresponding authors. The data are not publicly available due to clinical patient information.

## Supplementary Materials

The following supporting information can be downloaded at: https://www.mdpi.com/article/10.3390/diagnostics13010006/s1. Table S1: *p*-values of *post hoc* analyses comparing striatal SUVRs of types of atypical parkinsonism for regions with statistically significant differences in one-way ANOVA or Kruskal–Wallis tests.
