# Supplementary material for: Differences in Striatal Metabolism in [18F]FDG PET in Parkinson’s Disease and Atypical Parkinsonism
Source: Diagnostics (Basel). 2022 Dec 20;13(1):6. doi: 10.3390/diagnostics13010006 (PMC9818161; doi:10.3390/diagnostics13010006)
Supplement: Supplementary file 1 [file diagnostics-13-00006-s001.zip › diagnostics-2009744-supplementary.pdf]

Table S1. *P*-values of *post-hoc* analyses comparing striatal SUVRs of types of atypical parkinsonism for regions with statistically significant differences in one-way ANOVA or Kruskal-Wallis tests. Statistically significant differences are given in **bold**.

**Region**		**PSP - CBD**	**PSP - MSA**	**PSP - DLB**	**PSP - NC**	**CBD - MSA**	**CBD - DLB**	**CBD - NC**	**MSA - DLB**	**MSA - NC**	**DLB - NC**
Caudate	L	1.000	0.470	1.000	0.577	0.703	1.000	0.638	0.104	**0.035**	1.000
	R	1.000	0.839	1.000	0.124	1.000	1.000	0.166	0.148	**0.011**	1.000
Striatum	L	1.000	0.252	1.000	1.000	1.000	1.000	0.315	0.097	**0.037**	1.000
	R	1.000	0.337	1.000	0.130	1.000	1.000	0.052	0.170	**0.008**	0.532
	Whole	1.000	0.666	1.000	1.000	1.000	1.000	0.279	0.196	0.060	1.000
